# Hyperleptinemia Is a Risk Factor for the Development of Vascular Reactivity Impairment in Patients with Hypertension

**DOI:** 10.3390/medicina61122132

**Published:** 2025-11-28

**Authors:** I-Min Su, Li-Liang Chuang, Ji-Hung Wang, Bang-Gee Hsu

**Affiliations:** 1 Department of Anesthesiology, Dalin Tzu Chi Hospital, Buddhist Tzu Chi Medical Foundation, Chiayi 62247, Taiwan; 2 School of Medicine, Tzu Chi University, Hualien 97004, Taiwan; 3 Institute of Medical Sciences, Tzu Chi University, Hualien 97004, Taiwan; 4 Department of Internal Medicine, Hualien Tzu Chi Hospital, Buddhist Tzu Chi Medical Foundation, Hualien 97004, Taiwan; 5 Division of Critical Care Medicine, Hualien Tzu Chi Hospital, Buddhist Tzu Chi Medical Foundation, Hualien 97004, Taiwan; 6 Division of Cardiology, Buddhist Tzu Chi General Hospital, Hualien 97004, Taiwan; 7 Division of Nephrology, Hualien Tzu Chi Hospital, Buddhist Tzu Chi Medical Foundation, Hualien 97004, Taiwan

**Keywords:** age, leptin, hypertension, vascular reactivity index, endothelial dysfunction

## Abstract

*Background and Objectives*: Endothelial dysfunction represents an early indicator of cardiovascular disease in individuals with hypertension. Leptin, an adipokine that regulates vascular homeostasis and metabolism, has been linked to vascular impairment; however, its relationship with vascular reactivity in hypertensive patients remains unclear. *Materials and Methods*: A cross-sectional study was conducted involving 100 hypertensive patients recruited from the cardiovascular outpatient clinic of Hualien Tzu Chi Hospital between January and July 2021. Clinical profiles, anthropometric data, and laboratory results were collected. Endothelial function was evaluated through digital thermal monitoring, with vascular reactivity index (VRI) classified as poor (<1.0), intermediate (1.0–1.9), or good (≥2.0). The association between serum leptin levels and VRI was examined using correlation analysis, multivariable logistic regression, linear regression, and receiver operating characteristic (ROC) analysis. *Results*: Of the 100 participants, 10 (10%) exhibited poor VRI, 46 (46%) had intermediate VRI, and 44 (44%) had good VRI. Patients with impaired VRI were significantly older (*p* = 0.015), had higher waist circumference (*p* < 0.001), and showed higher serum leptin concentrations (*p* < 0.001). Multivariable logistic regression identified leptin as an independent factor associated with vascular reactivity dysfunction (OR = 1.096, 95% CI: 1.025–1.171, *p* = 0.007) and poor vascular reactivity (OR = 1.197, 95% CI: 1.034–1.387, *p* = 0.016). Serum leptin levels were negatively correlated with VRI (*r* = −0.408, *p* < 0.001), and stepwise linear regression confirmed leptin as an independent determinant of VRI (β = −0.296, *p* = 0.001). ROC analysis further demonstrated that leptin could predict vascular reactivity dysfunction (AUC = 0.724, 95% CI: 0.625–0.824, *p* < 0.001) and poor vascular reactivity (AUC = 0.770, 95% CI: 0.606–0.932, *p* = 0.0012). *Conclusions*: Higher serum leptin levels are independently associated with impaired vascular reactivity in hypertensive patients. Leptin may therefore serve as a potential biomarker for early impairment of vascular reactivity in this population.

## 1. Introduction

Hypertension remains one of the leading causes of cardiovascular morbidity and mortality worldwide, with its prevalence and burden continuing to rise [1]. Among the major modifiable risk factors for cardiovascular disease (CVD)—including elevated blood pressure, diabetes mellitus, dyslipidemia, and cigarette smoking—hypertension stands out as the most common and strongly associated determinant of adverse cardiovascular outcomes [2]. The vascular endothelium is essential for preserving vascular equilibrium and functional integrity by continuously releasing vasodilators that regulate vascular tone and resistance. A hallmark of endothelial dysfunction is diminished endothelium-mediated vasodilation along with pro-inflammatory and pro-thrombotic changes. This condition is widely recognized as an early marker and mediator of atherosclerosis [3]. Although blood pressure regulation involves complex renal, neural, and systemic mechanisms, endothelial dysfunction is increasingly viewed as a central pathological link connecting hypertension to elevated cardiovascular risk [4].

Leptin, an adipocytokine predominantly secreted by adipose tissue, is best known for regulating appetite and energy balance, with circulating concentrations rising markedly in obesity [5]. Emerging evidence indicates that leptin also exerts significant cardiovascular effects through receptors expressed in vascular tissues [6]. Elevated leptin has been associated with atherosclerosis [7,8] and identified as an independent associated factor of coronary artery disease (CAD) [9,10]. Beyond its metabolic actions, leptin promotes systemic inflammation by stimulating the secretion of pro-inflammatory mediators [11] and increases oxidative stress by enhancing the generation of reactive oxygen species [12], both of which contribute to vascular injury. Nonetheless, its specific role in endothelial function—an early event in vascular injury—remains unclear. Experimental studies have demonstrated that leptin can induce nitric oxide (NO)-mediated vasodilation under normal physiological conditions [13,14,15]. In contrast, in obesity or metabolic syndrome, it appears to impair endothelial responses, suggesting the emergence of leptin resistance [16,17]. Clinical findings have been similarly inconsistent, as associations between leptin and endothelial dysfunction often diminish after adjusting for body mass index (BMI) [18,19].

Although previous studies have examined the link between leptin and endothelial dysfunction, most relied on flow-mediated dilation or biochemical markers. To our knowledge, no prior study has evaluated this relationship using the vascular reactivity index (VRI) obtained from digital thermal monitoring in hypertensive patients [5,17,20]. Considering these uncertainties, this study aimed to determine whether elevated serum leptin levels are independently associated with vascular reactivity impairment, as assessed by the vascular reactivity index (VRI) obtained from digital thermal monitoring, in patients with hypertension.

## 2. Materials and Methods

### 2.1. Patients

Between January and July 2021, 100 patients with hypertension were recruited from the cardiovascular outpatient clinic at Buddhist Tzu Chi General Hospital in Hualien, Taiwan. The study protocol received ethical approval from the Research Ethics Committee of Hualien Tzu Chi Hospital (IRB108-219-A; 19 November 2019). Written informed consent was obtained from all participants prior to inclusion. Hypertension was defined according to the Eighth Joint National Committee (JNC 8) criteria as a systolic blood pressure (SBP) ≥ 140 mmHg, a diastolic blood pressure (DBP) ≥ 90 mmHg, or current use of antihypertensive medication within the previous two weeks. Blood pressure measurements were performed in the morning by trained personnel using a mercury sphygmomanometer and an appropriately sized cuff. Measurements were taken from the right arm after at least 10 min of rest, with three readings obtained at 5 min intervals and averaged for analysis. CAD was diagnosed when >50% stenosis was detected in any coronary segment on angiography. Diabetes mellitus (DM) was defined as a fasting plasma glucose level of ≥126 mg/dL or ongoing treatment with insulin or oral hypoglycemic agents. Patients were excluded if they had acute infection, malignancy, limb amputation, asthma, chronic obstructive pulmonary disease, acute coronary syndrome, or heart failure at the time of sampling, or if they declined to provide consent.

### 2.2. Anthropometric Analysis and Biochemical Determinations

All anthropometric assessments were performed in the morning after an overnight fast. Body weight and height were recorded to the nearest 0.5 kg and 0.5 cm, respectively. BMI was calculated as weight (kg) divided by height squared (m^2^). We measured waist circumference three times at the midpoint between the iliac crest and the lower rib margin, and recorded the average value. All blood samples were collected under standardized conditions to minimize preanalytical variability. Specifically, venous blood was drawn between 08:00 and 09:00 a.m. after an overnight fast of at least 10 h. Participants were instructed to refrain from strenuous exercise, smoking, alcohol consumption, and caffeine intake for 24 h prior to the visit. Upon arrival at the cardiovascular clinic, subjects were asked to rest in a seated position for 30 min in a quiet, temperature-controlled room prior to blood collection and anthropometric measurements. Blood sampling was performed immediately before vascular reactivity testing and anthropometric assessments. Venous blood samples (5 mL) were collected from each participant, centrifuged at 3000× *g* for 10 min, and stored at 4 °C within one hour for analysis. Serum levels of fasting glucose, albumin, blood urea nitrogen (BUN), creatinine, total cholesterol (TCH), triglycerides, high-density lipoprotein cholesterol (HDL-C), and low-density lipoprotein cholesterol (LDL-C) were determined using an automated analyzer (Siemens Advia 1800, Siemens Healthcare, Erlangen, Germany). Serum leptin concentrations were measured using a commercial enzyme immunoassay kit (SPI-Bio, Montigny-le-Bretonneux, France) [21]. Estimated glomerular filtration rate (eGFR) was calculated using the Chronic Kidney Disease Epidemiology Collaboration (CKD-EPI) equation.

### 2.3. Endothelial Function Measurements

Endothelial function was assessed using a digital thermal monitoring system (VENDYS-II; Endothelix, Inc., Houston, TX, USA), which is approved by the U.S. Food and Drug Administration. After resting supine for 30 min in a temperature-controlled room (22–24 °C), blood pressure cuffs were placed on the right upper arm, and skin temperature sensors were attached to the index fingers of both hands (left as control, right for occlusion). Following a 5 min stabilization period, the cuff was inflated to 50 mmHg above the SBP for 5 min and then rapidly deflated to induce reactive hyperemia. The vascular reactivity index (VRI) was automatically calculated as the maximal rebound in fingertip temperature relative to the baseline of zero reactivity during hyperemia. VRI values ranged from 0.0 to 3.5 and were categorized as poor (<1.0), intermediate (1.0–1.9), or good (≥2.0) vascular reactivity [22]. Vascular reactivity dysfunction was defined as the combination of intermediate and poor VRI categories (VRI < 2.0). To ensure measurement accuracy, the VENDYS-II system performs automatic calibration before each test according to the manufacturer’s protocol. All measurements were conducted by a single experienced operator to minimize inter-observer variability. Previous studies have demonstrated good reproducibility of digital thermal monitoring–derived indices and a significant correlation with flow-mediated dilation, supporting the validity of VRI as a noninvasive measure of endothelial function [22,23,24].

### 2.4. Statistical Analysis

Continuous data are summarized as mean ± standard deviation (SD). Data normality was assessed using the Kolmogorov–Smirnov test. Comparisons among groups (poor, intermediate, and good VRI) were conducted using one-way analysis of variance (ANOVA) for normally distributed variables and the Kruskal–Wallis test for non-normally distributed variables. Categorical variables were compared using the chi-square test. Post hoc pairwise comparisons were performed using Fisher’s protected t-test. Variables with skewed distributions (fasting glucose, TCH, triglycerides, LDL-C, BUN, and creatinine) were logarithmically transformed before analysis. Associations with VRI were first examined using simple linear regression for each candidate variable. Variables with *p* < 0.05 were subsequently entered into a forward stepwise multiple linear regression to identify independent associated factors. Multivariable logistic regression analysis was performed to assess the relationship between serum leptin levels and vascular reactivity dysfunction (VRI < 2.0) or poor vascular reactivity (VRI < 1.0) after adjusting for potential confounders. Receiver operating characteristic (ROC) curve analysis was used to evaluate the predictive performance of leptin for vascular reactivity dysfunction and poor vascular reactivity, and the corresponding area under the curve (AUC) values were calculated (MedCalc v22.019, Ostend, Belgium). To examine whether the association between serum leptin and vascular reactivity was consistent across subgroups, we conducted stratified analyses by sex (male, female) and by BMI categories (<27 vs. ≥27 kg/m^2^). Within each stratum, VRI was modeled as the dependent variable, and variables with *p* < 0.20 in the comparison between groups (poor, intermediate, and good VRI) were included (to prevent model overfit, 1 variable per 10 observed events was included, and BUN and creatinine were excluded due to their strong correlation with eGFR, which was retained as the renal function indicator). We evaluated calibration using the Hosmer–Lemeshow goodness-of-fit test and examined internal stability through nonparametric bootstrapping with 1000 resamples to assess potential model optimism and overfitting in vascular reactivity dysfunction. Multicollinearity was assessed with variance inflation factors (VIFs). We examined whether waist circumference mediated the association between leptin and VRI using a regression-based mediation approach (PROCESS macro for Statistical Package for the Social Sciences, version 4.2; model 4). All statistical analyses were two-tailed, and a *p* < 0.05 was considered statistically significant. All statistical analyses were performed using Statistical Package for the Social Sciences (version 25.0; IBM Corp., Armonk, NY, USA).

## 3. Results

The baseline clinical characteristics and antihypertensive treatments of the 100 patients with hypertension enrolled in the study are presented in Table 1. Among these participants, 10 (10.0%) exhibited a poor vascular reactivity index (VRI), 46 (46.0%) had an intermediate VRI, and 44 (44.0%) demonstrated a good VRI. Patients with lower VRI values were significantly older (*p* = 0.015), had higher waist circumference (*p* < 0.001), and had markedly higher serum leptin concentrations (*p* < 0.001) compared with those showing better vascular reactivity. No significant differences were found among the groups in terms of sex, smoking status, diabetes mellitus (DM), coronary artery disease (CAD), body mass index (BMI), or the use of antihypertensive or lipid-lowering medications.

Multivariable logistic regression analysis revealed that serum leptin levels were independently and positively associated with vascular reactivity dysfunction or poor vascular reactivity. After adjusting for age, DM, smoking status, BMI, waist circumference, blood pressure, renal function, lipid profile, and albumin levels, patients with vascular reactivity dysfunction (combined intermediate and poor VRI groups) had an odds ratio (OR) of 1.096 (95% CI: 1.025–1.171; *p* = 0.007) compared with those in the good VRI group. Similarly, individuals with poor vascular reactivity demonstrated an OR of 1.197 (95% CI: 1.034–1.387; *p* = 0.016) (Table 2). VIFs for all predictor variables were <3.5, so no additional collinearity diagnostics were conducted in the multivariable analysis.

**Figure 1:**
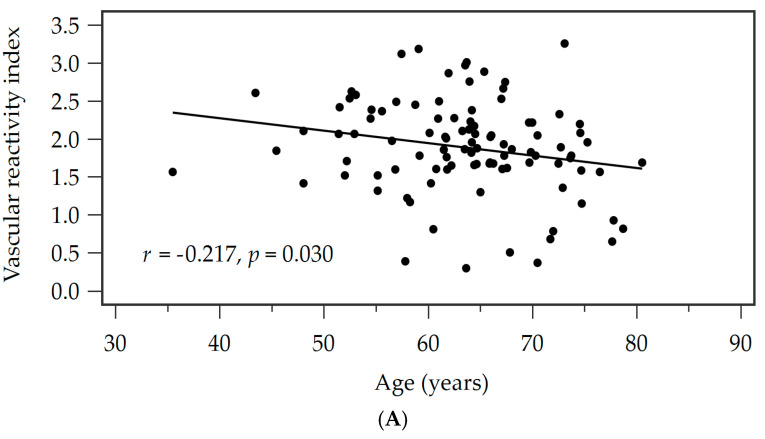

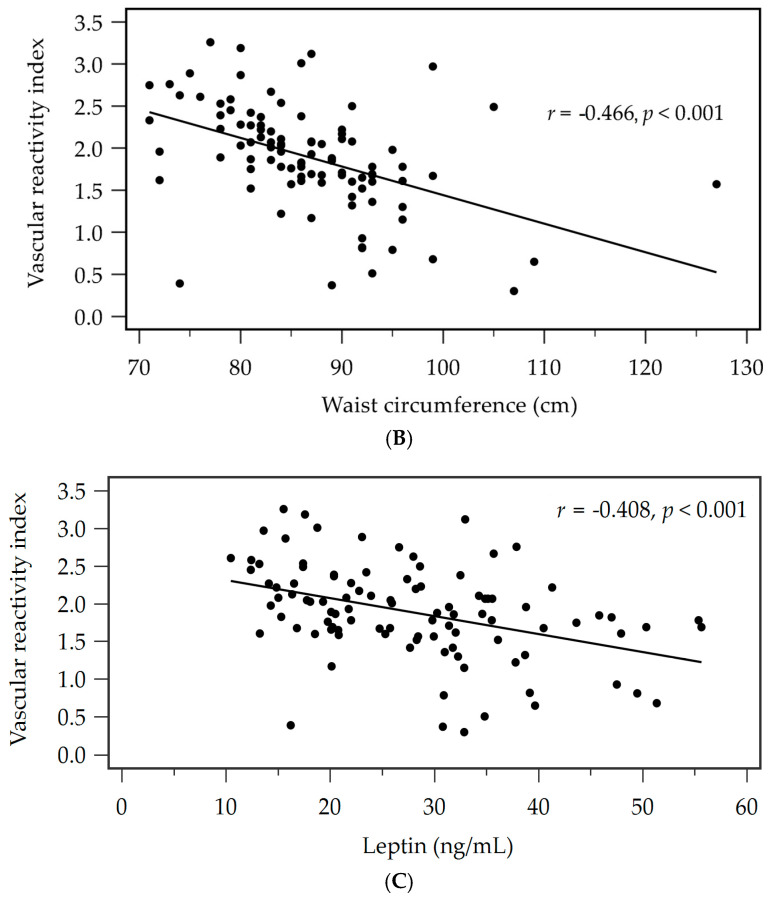
Associations between vascular reactive index (VRI) and (**A**) age, (**B**) waist circumference, and (**C**) leptin levels in hypertensive patients.

Correlation analysis demonstrated that both increasing age (*r* = −0.217, *p* = 0.030), higher waist circumference (*r* = −0.466, *p* < 0.001), and higher serum leptin levels (*r* = −0.408, *p* < 0.001) were significantly and inversely correlated with VRI values (Table 3). In stepwise linear regression analysis, age (β = −0.178, adjusted R^2^ change = 0.025, *p* = 0.036), waist circumference (β = −0.413, adjusted R^2^ change = 0.209, *p* < 0.001), and serum leptin concentration (β = −0.296, adjusted R^2^ change = 0.098, *p* = 0.001) were identified as independent factors associated with impaired vascular reactivity. The relationships between VRI and age (Figure 1A), waist circumference (Figure 1B), and leptin levels (Figure 1C) are illustrated in the corresponding scatter plots.

We further stratified the analysis by BMI (<27 vs. ≥27 kg/m^2^) and treated VRI as a continuous outcome, and variables with *p* < 0.2 at Table 1 were entered into a forward stepwise multiple linear regression, adjusting for DM, smoking, age, height, body weight, waist circumference, eGFR, HDL-C, and leptin. Waist circumference was inversely associated with VRI among participants with BMI < 27 kg/m^2^ (unstandardized coefficient [B] = −0.045, β = −0.490, *p* < 0.001), accounting for 22.5% of the variance (adjusted R^2^ = 0.225). In those with BMI ≥ 27 kg/m^2^, waist circumference remained a significant correlate of lower VRI (B = −0.024, β = −0.444, *p* < 0.001; adjusted R^2^ = 0.211). After leptin was added to the model for individuals with a BMI ≥ 27 kg/m^2^, leptin was found to be independently and inversely related to VRI (B = −0.022, β = −0.438, *p* < 0.001), and the model’s performance improved (adjusted R^2^ = 0.393). These findings suggest that, particularly in patients with higher BMI, leptin provides incremental explanatory value for vascular reactivity beyond central adiposity (Supplementary Table S1).

In sex-stratified analyses (Supplementary Table S2), both waist circumference and leptin were independently associated with VRI in men. Among men, greater waist circumference was related to lower VRI (B = −0.027, β = −0.383, *p* < 0.001), and leptin contributed additional variance beyond adiposity (B = −0.017, β = −0.308, *p* = 0.002), improving the model from an adjusted R^2^ of 0.172 to 0.257 (*p* = 0.002). In women, leptin alone showed a significant inverse association with VRI (B = −0.042, β = −0.588, *p* = 0.010), accounting for 30.4% of the variance (adjusted R^2^ = 0.304) despite the small sample size. These findings suggest that leptin maintains an independent relationship with vascular reactivity after accounting for central adiposity, particularly in men, and that the signal remains detectable in women.

ROC curve analysis revealed that serum leptin was a significantly associated factor of impaired vascular reactivity. The AUC was 0.724 (95% CI: 0.625–0.824; *p* < 0.001) for vascular reactivity dysfunction and 0.770 (95% CI: 0.606–0.932; *p* = 0.0012) for poor vascular reactivity. According to the Youden index, the optimal serum leptin cutoff value for predicting vascular reactivity dysfunction was 28.70 ng/mL, yielding a sensitivity of 60.7%, a specificity of 79.6%, a positive predictive value (PPV) of 79.1%, and a negative predictive value (NPV) of 61.4%. For predicting poor vascular reactivity, the optimal cutoff was 30.25 ng/mL, with a sensitivity of 90.0%, specificity of 65.6%, PPV of 22.5%, and NPV of 98.3% (Table 4).

In bootstrap internal validation (1000 resamples) of multivariable logistic regression for vascular reactivity dysfunction, including leptin, waist circumference, age, DM, smoking, height, body weight, HDL-C, and eGFR (*p* < 0.20 in Table 1). Serum leptin (B = 0.092, bias-corrected and accelerated [BCa] 95% CI: 0.009 to 0.361, *p* = 0.004) and waist circumference (B = 0.147, BCa 95% CI: 0.027 to 0.825, *p* < 0.001) remained significant predictors of vascular reactivity dysfunction, with BCa 95% CIs that did not include zero, indicating stable estimates (Table 5). In contrast, the effects of DM, smoking, body weight, HDL-C, and eGFR were attenuated and their BCa CIs crossed zero, suggesting limited stability of these covariates, likely due to the small number of events.

Model calibration was acceptable (Hosmer–Lemeshow χ^2^ = 6.102, *p* = 0.636) for vascular reactivity dysfunction. Calibration was evaluated by plotting observed versus predicted vascular reactivity dysfunction across deciles of predicted risk. The plot showed acceptable agreement in the higher-risk deciles, although the model tended to underpredict risk in the lowest deciles and slightly overpredict around the middle decile (Supplementary Figure S1). Decision curve analysis based on the predicted probabilities from the leptin-based logistic model showed a positive net benefit over a wide range of threshold probabilities (approximately 0.05–0.65) compared with both the treat-all and treat-none strategies, indicating potential clinical usefulness of leptin for identifying patients with vascular reactivity dysfunction (Supplementary Figure S2).

Using PROCESS version 4 for SPSS (model 4) with 5000 bootstrap resamples, we examined whether waist circumference mediated the association between leptin and VRI, while adjusting for age, height, body weight, eGFR, HDL-C, diabetes history, and smoking (*p* < 0.2, as shown in Table 1). Leptin showed a significant inverse association with VRI in the total-effect model (B = −0.022, 95% CI: −0.034 to −0.010, *p* = 0.0004). In the mediator model, higher leptin levels were associated with a larger waist circumference, but this path did not reach statistical significance after covariate adjustment (B = 0.142, *p* = 0.113). In the outcome model, both leptin (B = −0.018, *p* = 0.001) and waist circumference (B = −0.029, *p* < 0.001) remained independent predictors of lower VRI. The estimated indirect effect of leptin on VRI through waist circumference was small (effect = −0.0041) and its 95% bootstrap confidence interval crossed zero (−0.0113 to 0.0008). This suggests that leptin was associated with impaired vascular reactivity independent of waist circumference. Instead, the inverse association between leptin and VRI persisted after adjustment for waist circumference and other covariates (Supplementary Table S3).

## 4. Discussion

In this study of hypertensive patients, we found that elevated serum leptin levels were significantly associated with impaired vascular reactivity. Individuals with lower VRI values were older, had higher waist circumference, and exhibited markedly higher leptin concentrations. Multivariate analyses confirmed leptin as an independent determinant of endothelial dysfunction, even after adjusting for conventional cardiovascular risk factors. Correlation and regression analyses further demonstrated inverse relationships between VRI and both age and leptin, emphasizing their contributory roles in vascular impairment. Moreover, ROC curve analysis showed that serum leptin effectively predicted vascular reactivity dysfunction, with good discriminatory accuracy and clinically meaningful cutoff values. Collectively, these findings extend previous evidence by demonstrating that elevated serum leptin levels are associated with vascular reactivity impairment in hypertensive patients, as assessed using digital thermal monitoring.

The vascular endothelium plays a central role in maintaining vascular homeostasis by releasing mediators such as NO, which regulate vasodilation, angiogenesis, and coagulation [25]. Reduced NO bioavailability or increased degradation is a hallmark of endothelial dysfunction [26], which is largely influenced by oxidative stress, persistent inflammation, and traditional cardiovascular risk factors, including hypertension, diabetes, obesity, dyslipidemia, and excess dietary sodium [27,28,29]. Age is another key determinant of endothelial impairment; older adults often display diminished endothelium-dependent dilation even in the absence of overt disease [30]. In support of this, Naghavi et al. analyzed digital thermal monitoring data from 6084 patients and found an inverse association between age and VRI, identifying age as a significant, though relatively weak, predictor [22]. Waist circumference is a cardiovascular risk indicator that is independent of overall adiposity as measured by BMI [31]. Consistent with these observations, our study also demonstrated that advancing age and higher waist circumference were correlated with lower VRI values.

Beyond its established role in energy regulation, leptin contributes to endothelial dysfunction through multiple pathophysiological mechanisms [32]. Experimental evidence suggests that elevated leptin promotes oxidative stress [33], inflammation [34], and reduced NO bioavailability [35]. Moreover, leptin resistance—commonly seen in obesity and metabolic syndrome—further impairs endothelium-dependent vasodilation [36]. Clinical studies have reported similar associations between plasma leptin and vascular reactivity, though some were attenuated after adjustment for BMI [18,19], highlighting the potential confounding influence of adiposity. In our hypertensive cohort, leptin levels were significantly higher among patients with poorer vascular reactivity. Multivariate logistic regression confirmed leptin as an independent predictor of both vascular reactivity dysfunction and poor vascular reactivity, while stepwise regression revealed an inverse association with VRI. ROC analysis demonstrated moderate discriminatory ability for both vascular reactivity dysfunction (AUC = 0.724) and poor vascular reactivity (AUC = 0.770). These findings support the role of hyperleptinemia as a contributor to endothelial impairment and a potential early biomarker of impaired vascular reactivity in hypertension.

The mechanisms underlying leptin-induced endothelial dysfunction involve several interconnected molecular pathways. Chronic hyperleptinemia disrupts the phosphoinositide 3-kinase (PI3K)/protein kinase B (Akt)/endothelial nitric oxide synthase (eNOS) pathway, essential for NO synthesis and endothelium-dependent vasodilation [37]. In the context of leptin resistance—frequently observed in obesity and metabolic syndrome—leptin’s normal vasodilatory effects are blunted, while its mitogen-activated protein kinase (MAPK)-mediated vasoconstrictive and pro-inflammatory actions are preserved [19,38]. This selective resistance promotes an imbalance that favors vasoconstriction, oxidative stress, and vascular inflammation. Leptin also enhances endothelial dysfunction by stimulating endothelin-1 production, a potent vasoconstrictor, while simultaneously reducing NO bioavailability through the generation of reactive oxygen species via NADPH oxidase activation [39,40]. The resulting oxidative stress not only depletes NO by forming peroxynitrite but also leads to eNOS uncoupling, converting the enzyme from an NO producer into a superoxide generator [41]. These molecular disturbances impair both local and systemic endothelial function. In addition, hyperleptinemia stimulates sympathetic nervous system activity and increases angiotensin II levels, further aggravating endothelial injury and hypertension [42,43].

Although our cohort comprised exclusively patients with hypertension, the observed inverse association between leptin and endothelial function is biologically plausible beyond the hypertensive setting. Prior studies have linked hyperleptinemia to impaired endothelial reactivity in overweight patients with type 2 diabetes [5] and in community-dwelling elderly without overt hypertension [19]. Experimental work further shows that chronic leptin excess promotes oxidative stress, attenuates NO bioavailability, and induces endothelial dysfunction, particularly under conditions of adiposity and metabolic dysregulation [16,17]. Together, these data suggest that the leptin–endothelial axis may represent a broader physiological phenomenon relevant across cardiometabolic phenotypes rather than a relationship confined to hypertension. In our internal stratified analyses, the leptin–VRI association remained directionally consistent across sex and BMI categories. Notably, leptin contributed additional explanatory value beyond central adiposity among participants with higher BMI, and this association was still observable in women, despite the smaller number of female participants in the cohort. Moreover, mediation analysis did not support waist circumference as a major mediator of the leptin–VRI association, suggesting that leptin may relate to endothelial impairment through pathways that only partly overlap with central adiposity. These findings increase confidence that the association is not solely an artifact of adiposity confounding within this hypertensive population.

The clinical implications of these findings extend beyond statistical associations and may inform cardiovascular risk management. Based on the identified cutoff values (28–32 ng/mL), serum leptin measurement could potentially be integrated into cardiovascular risk assessment frameworks. Although formal guidelines for leptin-based risk stratification in hypertension are not yet established, our findings suggest a framework for future validation: (1) identifying high-risk hypertensive patients (e.g., those with BMI > 30 kg/m^2^, diabetes, or multiple metabolic risk factors) who might benefit from leptin testing; (2) implementing intensified monitoring and aggressive risk modification for those with markedly elevated leptin levels; and (3) applying evidence-based interventions known to reduce leptin levels and improve vascular health [44]. Lifestyle interventions remain the cornerstone of managing hyperleptinemia and endothelial dysfunction. Aerobic exercise has been shown to lower leptin concentrations by 20–30% and improve endothelial function through enhanced insulin and leptin sensitivity [45]. Dietary interventions—particularly adherence to a Mediterranean diet or intermittent fasting—also demonstrate beneficial effects on leptin regulation and vascular health [46]. Pharmacologically, metformin has been shown to enhance leptin sensitivity and endothelial function independent of weight loss, making it a promising option for hypertensive patients with metabolic abnormalities [47]. Furthermore, glucagon-like peptide-1 receptor agonists reduce leptin levels via weight loss and improve endothelial function through leptin-independent mechanisms [48]. Integrating leptin measurements into cardiovascular risk calculators may therefore enhance the early detection and prevention of cardiovascular disease. For example, patients with leptin levels exceeding 30 ng/mL and intermediate VRI (1.0–1.9) could benefit from more frequent cardiovascular monitoring (e.g., every six months instead of annually) and earlier initiation of preventive therapies.

Certain limitations of this investigation should be recognized. First, the cross-sectional design precludes causal inference between hyperleptinemia and endothelial dysfunction. Second, as the study was conducted at a single center with hypertensive patients, generalizability to broader populations may be limited. Third, all participants had a mean body mass index above the normal range, consistent with the high prevalence of overweight and obesity among hypertensive individuals. Although BMI was similar across VRI categories and was adjusted for in multivariable analyses, excess adiposity may have contributed to baseline endothelial impairment and hyperleptinemia, potentially attenuating intergroup differences and limiting the extrapolation to lean or normotensive populations. Fourth, the small number of participants with poor VRI (n = 10) could have reduced the power of subgroup analyses. Fifth, although multiple confounding factors were adjusted for, the possibility of residual confounding cannot be entirely excluded. Unmeasured variables such as dietary habits, physical activity, systemic inflammation, insulin resistance, or the use of antidiabetic medications may still have influenced the observed associations. In particular, while metformin and other antidiabetic agents have been reported to enhance leptin sensitivity and endothelial function, data from these medications were not available in our dataset; therefore, these considerations are discussed solely in the context of prior evidence rather than findings from the present study. Finally, leptin levels were measured at a single time point, which prevented the assessment of temporal variation. Future studies should therefore employ larger, multicenter cohorts with longitudinal follow-up to clarify causal relationships and determine whether leptin can predict long-term vascular outcomes.

## 5. Conclusions

In summary, this study demonstrated that elevated serum leptin levels are independently associated with vascular reactivity impairment in patients with hypertension, as assessed by digital thermal monitoring. These findings extend previous evidence linking leptin to endothelial dysfunction by confirming this association in hypertensive individuals using a noninvasive, automated VRI. Further studies are warranted to validate the clinical utility of VRI-based assessment for vascular risk stratification in this population.

## Figures and Tables

**Table 1:** Clinical characteristics by vascular reactivity index category measured via digital thermal monitoring among 100 hypertensive patients.

Characteristics	All Participants (*n* = 100)	Good Vascular Reactivity (*n* = 44)	Intermediate Vascular Reactivity (*n* = 46)	Poor Vascular Reactivity (*n* = 10)	*p* Value
Age (years)	63.52 ± 8.26	61.61 ± 7.20	63.98 ± 8.79	66.79 ± 7.35	0.015 *
Height (cm)	163.89 ± 7.40	162.30 ± 7.63	165.51 ± 7.44	163.50 ± 4.65	0.118
Body weight (kg)	73.39 ± 11.52	71.03 ± 9.35	76.27 ± 13.53	70.57 ± 6.98	0.069
Body mass index (kg/m^2^)	27.27 ± 3.47	26.96 ± 3.05	27.76 ± 4.09	26.34 ± 1.44	0.373
Waist circumference (cm)	86.82 ± 8.53	82.84 ± 6.62	89.02 ± 8.17	94.20 ± 9.76	<0.001 *
Vascular reactivity index	1.89 ± 0.62	2.42 ± 0.35	1.66 ± 0.21	0.62 ± 0.22	<0.001 *
Systolic blood pressure (mmHg)	135.17 ± 18.66	136.68 ± 16.86	134.63 ± 20.14	131.00 ± 20.29	0.666
Diastolic blood pressure (mmHg)	79.96 ± 11.05	81.55 ± 10.04	78.98 ± 12.00	77.50 ± 10.78	0.418
Total cholesterol (mg/dL)	139.50 (141.00–178.00)	160.00 (141.00–176.75)	156.50 (135.75–187.50)	172.00 (154.00–189.50)	0.351
Triglyceride (mg/dL)	141.00 (104.50–206.00)	136.00 (101.25–205.50)	138.00 (110.00–204.00)	174.00 (83.75–217.00)	0.892
HDL-C (mg/dL)	46.44 ± 10.29	47.57 ± 10.14	44.39 ± 10.03	50.90 ± 10.95	0.120
LDL-C (mg/dL)	87.00 (71.00–106.00)	87.00 (70.25–97.00)	85.50 (69.75–112.25)	96.00 (79.50–113.25)	0.446
Fasting glucose (mg/dL)	112.00 (92.25–152.25)	115.00 (95.50–153.75)	112.00 (92.75–155.75)	98.50 (87.25–136.75)	0.297
Albumin (mg/dL)	4.37 ± 0.23	4.41 ± 0.25	4.34 ± 0.19	4.30 ± 0.31	0.239
Blood urea nitrogen (mg/dL)	17.00 (14.00–20.00)	16.50 (13.00–19.00)	17.00 (14.00–22.25)	18.50 (13.75–23.50)	0.164
Creatinine (mg/dL)	1.00 (0.83–1.10)	0.90 (0.80–1.10)	1.00 (0.90–1.13)	1.00 (0.90–1.10)	0.160
eGFR (mL/min)	80.76 ± 22.44	86.27 ± 23.71	77.34 ± 20.13	72.21 ± 23.20	0.074
Leptin (ng/mL)	27.82 ± 10.65	23.02 ± 8.08	30.36 ± 10.75	37.27 ± 10.60	<0.001 *
Male, *n* (%)	82 (82.0)	35 (79.5)	40 (87.0)	7 (70.0)	0.383
Diabetes mellitus, *n* (%)	48 (48.0)	23 (52.3)	18 (39.1)	7 (70.0)	0.156
Coronary artery disease, *n* (%)	72 (72.0)	32 (72.7)	34 (73.9)	6 (60.0)	0.667
Smoking, *n* (%)	19 (19.0)	12 (27.3)	5 (10.9)	2 (20.0)	0.140
ACE inhibitor use, *n* (%)	20 (20.0)	9 (20.5)	10 (21.7)	1 (10.0)	0.669
ARB use, *n* (%)	51 (51.0)	24 (54.5)	21 (45.7)	6 (60.0)	0.585
β-blocker use, *n* (%)	43 (43.0)	17 (38.6)	21 (45.7)	5 (50.0)	0.714
CCB use, *n* (%)	45 (45.0)	21 (47.7)	21 (45.7)	3 (30.0)	0.592
Statin use, *n* (%)	74 (74.0)	31 (70.5)	36 (78.3)	7 (70.0)	0.669
Fibrate use, *n* (%)	7 (7.0)	4 (9.1)	2 (4.3)	1 (10.0)	0.628

Continuous variables are summarized as mean ± standard deviation and compared using one-way ANOVA. Non-normally distributed data are expressed as median (interquartile range) and analyzed with the Kruskal–Wallis test. Categorical variables are presented as numbers (percentages) and evaluated using the chi-square test. HDL-C, high-density lipoprotein cholesterol; LDL-C, low-density lipoprotein cholesterol; eGFR, estimated glomerular filtration rate; ACE, angiotensin-converting enzyme; ARB, angiotensin II receptor blocker; CCB, calcium-channel blocker. * *p* < 0.05 indicates statistical significance.

**Table 2:** Adjusted logistic regression analyses of vascular reactivity dysfunction and poor vascular reactivity among 100 hypertensive patients.

Model	Leptin (per 1 ng/mL of Increase) for Vascular Reactivity Dysfunction		Leptin (per 1 ng/mL of Increase) for Poor Vascular Reactivity	
	OR (95% CI)	*p* Value	OR (95% CI)	*p* Value
Crude model	1.098 (1.045–1.153)	<0.001 *	1.092 (1.025–1.162)	0.006 *
Adjusted model	1.096 (1.025–1.171)	0.007 *	1.197 (1.034–1.387)	0.016 *

Adjusted model: adjusted for sex, age, diabetes mellitus, smoking, body mass index, waist circumference, systolic blood pressure, diastolic blood pressure, eGFR, albumin, total cholesterol, triglyceride, HDL-C, and LDL-C. HDL-C, high-density lipoprotein cholesterol; LDL-C, low-density lipoprotein cholesterol; eGFR, estimated glomerular filtration rate; OR, odds ratio; CI, confidence interval. * *p* < 0.05 was considered statistically significant.

**Table 3:** Associations between the vascular reactivity index and clinical variables using simple and multiple linear regression analyses in 100 hypertensive patients.

Variables	Vascular Reactivity Index				
	Simple Regression		Multivariable Regression		
	*r*	*p* Value	Beta	Adjusted R^2^ Change	*p* Value
Age (years)	−0.217	0.030 *	−0.178	0.025	0.036 *
Height (cm)	−0.069	0.494	–	–	–
Body weight (kg)	−0.095	0.345	–	–	–
Body mass index (kg/m^2^)	−0.061	0.544	–	–	–
Waist circumference (cm)	−0.466	<0.001 *	−0.413	0.209	<0.001 *
SBP (mmHg)	0.089	0.379	–	–	–
DBP (mmHg)	0.123	0.222	–	–	–
Log-TCH (mg/dL)	−0.163	0.104	–	–	–
Log-Triglyceride (mg/dL)	−0.044	0.663	–	–	–
HDL-C (mg/dL)	−0.019	0.847	–	–	–
Log-LDL-C (mg/dL)	−0.112	0.268	–	–	–
Log-Glucose (mg/dL)	0.143	0.157	–	–	–
Albumin (mg/dL)	0.167	0.096	–	–	–
Log-BUN (mg/dL)	−0.169	0.092	–	–	–
Log-Creatinine (mg/dL)	−0.124	0.220	–	–	–
eGFR (mL/min)	0.153	0.129	–	–	–
Leptin (ng/mL)	−0.408	<0.001 *	−0.296	0.098	0.001 *

Data for TCH, triglycerides, LDL-C, fasting glucose, blood urea nitrogen, and creatinine exhibited a skewed distribution; therefore, they were log-transformed before analysis. Simple linear regression was performed for each variable. Variables with *p* < 0.05 were entered into a forward stepwise multiple linear regression to identify independent correlates of VRI (adopted factors: age, waist circumference, leptin). TCH, total cholesterol; SBP, systolic blood pressure; DBP, diastolic blood pressure; HDL-C, high-density lipoprotein cholesterol; LDL-C, low-density lipoprotein cholesterol; BUN, blood urea nitrogen; eGFR, estimated glomerular filtration rate. * *p* < 0.05 was considered statistically significant.

**Table 4:** Receiver operating characteristic (ROC) analysis of serum leptin for vascular reactivity dysfunction (combined intermediate and poor vascular reactivity) and for poor vascular reactivity alone.

	**Vascular Reactivity Dysfunction**						
	AUC (95% CI)	*p* Value	Cut-Off	Sen (%)	Spe (%)	PPV (%)	NPV (%)
Leptin (ng/mL)	0.724 (0.625–0.824)	<0.001 *	28.70	60.71	79.55	79.07	61.40
	**Poor Vascular Reactivity**						
	AUC (95% CI)	*p* Value	Cut-Off	Sen (%)	Spe (%)	PPV (%)	NPV (%)
Leptin (ng/mL)	0.770 (0.606–0.932)	0.0012 *	30.25	90.0	65.56	22.50	98.33

AUC, area under the curve; 95% CI, 95% confidence interval; Sen, sensitivity; Spe, specificity; PPV, positive predictive value; NPV, negative predictive value. * *p* < 0.05 was considered statistically significant.

**Table 5:** Multivariable logistic regression with bootstrap resampling (B = 1000) of the factors correlated to vascular reactivity dysfunction in 100 hypertensive patients.

Variables	B	BCa 95% CI	*p* Value
Leptin, ng/mL	0.092	0.009, 0.361	0.004
Waist circumference, cm	0.147	0.027, 0.825	<0.001
Age, year	0.099	−0.014, 0.456	0.027
Diabetes, present	−0.349	−0.2.165, 1.047	0.591
Smoking, present	−0.863	−2.804, 0.516	0.237
Height, cm	0.097	−0.060, 0.439	0.058
Body weight, kg	0.018	−0.076, 0.141	0.581
HDL-C, mg/dL	−0.024	−0.112, 0.039	0.445
eGFR, mL/min	−0.009	−0.062, 0.026	0.594

Data were analyzed using multivariable logistic regression with 1000 bootstrap resamples. Candidate variables (diabetes mellitus, smoking, age, height, body weight, waist circumference, eGFR, HDL-C, and leptin) were entered simultaneously. Coefficients (B) are log-odds estimates from the fitted model. Bias-corrected and accelerated (BCa) 95% confidence intervals are based on 1000 bootstrap samples. BCa, bias-corrected and accelerated; CI, confidence interval; HDL-C, high-density lipoprotein cholesterol; eGFR, estimated glomerular filtration rate.

## Data Availability

The corresponding author can provide the data used in this study upon request. The data are not publicly available due to ethical restrictions and institutional policies protecting participant privacy (IRB108-219-A).

## Supplementary Materials

The following supporting information can be downloaded at: https://www.mdpi.com/article/10.3390/medicina61122132/s1, Table S1: Linear regression of VRI on waist circumference and leptin, stratified by BMI; Table S2: Linear regression of VRI on waist circumference and leptin, stratified by gender; Table S3: Mediation analysis of the association between leptin and VRI through waist circumference; Figure S1: Calibration plot for vascular reactivity dysfunction; Figure S2: Decision curve analysis for the leptin-based logistic model predicting vascular reactivity dysfunction.

## Abbreviations

The following abbreviations are used in this manuscript:

AUC	Areas under the curve
BCa	Bias-corrected and accelerated
BMI	Body mass index
BUN	Blood urea nitrogen
CAD	Coronary artery disease
CVD	Cardiovascular disease
DBP	Diastolic blood pressure
DM	Diabetes mellitus
eGFR	Estimated glomerular filtration rate
eNOS	Endothelial nitric oxide synthase
HDL-C	High-density lipoprotein cholesterol
LDL-C	Low-density lipoprotein cholesterol
NO	Nitric oxide
PI3K	Phosphoinositide 3-kinase
ROC	Receiver operating characteristic
SBP	Systolic blood pressure
TCH	Total cholesterol
VRI	Vascular reactivity index
