# Supplementary material for: Hyperleptinemia Is a Risk Factor for the Development of Vascular Reactivity Impairment in Patients with Hypertension
Source: Medicina (Kaunas). 2025 Nov 28;61(12):2132. doi: 10.3390/medicina61122132 (PMC12735040; doi:10.3390/medicina61122132)
Supplement: Supplementary file 1 [file medicina-61-02132-s001.zip › medicina-3944249-supplementary.pdf]

Supplementary Table S1. Linear regression of VRI on waist circumference and leptin, stratified by BMI.

BMI stratum	Predictor	B	SE	β	Adjusted R^2 Change	*p* Value
BMI < 27 (*n* = 53)	Waist circumference	−0.045	0.011	−0.490	0.225	<0.001*
	Constant	5.749	0.983	–		<0.001*
Model statistics: R = 0.490; R^2 = 0.240; adjusted R^2 = 0.225; SEE = 0.607; $p < 0.001$*						
BMI ≥ 27 (*n* = 47)	Waist circumference	−0.024	0.006	−0.444	0.211	<0.001*
	Leptin	−0.022	0.006	−0.438	0.182	<0.001*
	Constant	4.705	0.565	–		<0.001*
Model statistics: R = 0.648; R^2 = 0.420; adjusted R^2 = 0.393; SEE = 0.415; $p < 0.001$*						

Variables with $p < 0.2$ at Table 1 were entered into a forward stepwise multiple linear regression to identify independent correlates of VRI (adjusted factors: diabetes mellitus, smoking, age, height, body weight, waist circumference, eGFR, HDL-C, leptin). VRI, vascular reactivity index; BMI, body mass index; eGFR, estimated glomerular filtration rate; HDL-C, high-density lipoprotein-cholesterol; SEE, standard error of the estimate. B denotes the unstandardized regression coefficient, β the standardized coefficient. * $p < 0.05$ was considered statistically significant.

Supplementary Table S2. Linear regression of VRI on waist circumference and leptin, stratified by gender.

Sex	Predictor	B	SE	β	Adjusted R^2 Change	*p* Value
Men (*n* = 81)	Waist circumference	−0.027	0.007	−0.383	0.172	<0.001*
	Leptin	−0.017	0.005	−0.308	0.085	0.002*
	Constant	4.742	0.599	–		<0.001*
Model fit: R = 0.525; R^2 = 0.275; adjusted R^2 = 0.257; SEE = 0.509; *p* = 0.002*p*						
Women (*n* = 18)	Leptin	−0.042	0.015	−0.588	0.304	0.010*
	Constant	3.015	0.426	–		<0.001*
Model fit: R = 0.588; R^2 = 0.345; adjusted R^2 = 0.304; SEE = 0.645; *p* = 0.010*						

Variables with $p < 0.2$ at Table 1 were entered into a forward stepwise multiple linear regression to identify independent correlates of VRI (adjusted factors: diabetes mellitus, smoking, age, height, body weight, waist circumference, eGFR, HDL-C, leptin). VRI, vascular reactivity index; eGFR, estimated glomerular filtration rate; HDL-C, high-density lipoprotein-cholesterol; SEE, standard error of the estimate. B denotes the unstandardized regression coefficient, β the standardized coefficient. * $p < 0.05$ was considered statistically significant.

Supplementary Table S3. Mediation analysis of the association between leptin and VRI through waist circumference (PROCESS model 4, 5,000 bootstrap samples).

Outcome/model	Predictor	B	SE	β	95% CI for B	*p* Value
Mediator model (M = waist circumference)						
	Leptin	0.142	0.089	0.177	−0.034 to 0.318	0.113
	Age	−0.000	0.111	0.000	−0.220 to 0.220	0.998
	Height	−0.015	0.141	−0.013	−0.295 to 0.265	0.917
	Body weight	0.224	0.101	0.302	0.024 to 0.424	0.028*
	eGFR	−0.018	0.043	−0.047	−0.103 to 0.067	0.677
	HDL-C	0.121	0.092	0.146	−0.062 to 0.304	0.192
	Diabetes history	0.421	1.745	0.025	−3.05 to 3.89	0.810
	Smoking	−1.244	2.224	−0.058	−5.66 to 3.17	0.577
	Constant	64.74	23.33	–	18.40 to 111.07	0.007
	Model fit: R = 0.337; R^2 = 0.113; MSE = 70.24; p = 0.186					
Outcome model (Y = VRI)						
	Leptin (direct effect)	−0.018	0.006	−0.313	−0.029 to −0.007	0.001*
	Waist circumference	−0.029	0.007	−0.395	−0.042 to −0.016	<0.001*
	Age	−0.014	0.007	−0.190	−0.028 to −0.001	0.041*
	Height	−0.005	0.009	−0.063	−0.023 to 0.012	0.551
	Body weight	−0.002	0.006	−0.029	−0.014 to 0.011	0.808
	eGFR	−0.001	0.003	−0.032	−0.006 to 0.004	0.738
	HDL-C	−0.002	0.006	−0.039	−0.014 to 0.009	0.683
	Diabetes history	−0.011	0.109	−0.009	−0.227 to 0.205	0.919
	Smoking	0.079	0.139	0.050	−0.197 to 0.355	0.571
	Constant	6.96	1.51	–	3.95 to 9.97	<0.001*
	Model fit: R = 0.601; R^2 = 0.361; MSE = 0.273; p < 0.001*					
Total effect (without mediator)	Leptin	−0.022	0.006	−0.383	−0.034 to −0.010	0.0004*
Indirect effect (Leptin → Waist → VRI)	Effect = −0.0041	Boot SE = 0.0031	Boot 95% CI = −0.0113 to 0.0008			

VRI, vascular reactivity index; eGFR, estimated glomerular filtration rate; HDL-C, high-density lipoprotein-cholesterol; CI, confidence interval. Indirect effect CI includes zero, indicating the mediation was not statistically significant. * $p < 0.05$ was considered statistically significant.

Supplementary Figure S1. Calibration plot for vascular reactivity dysfunction.

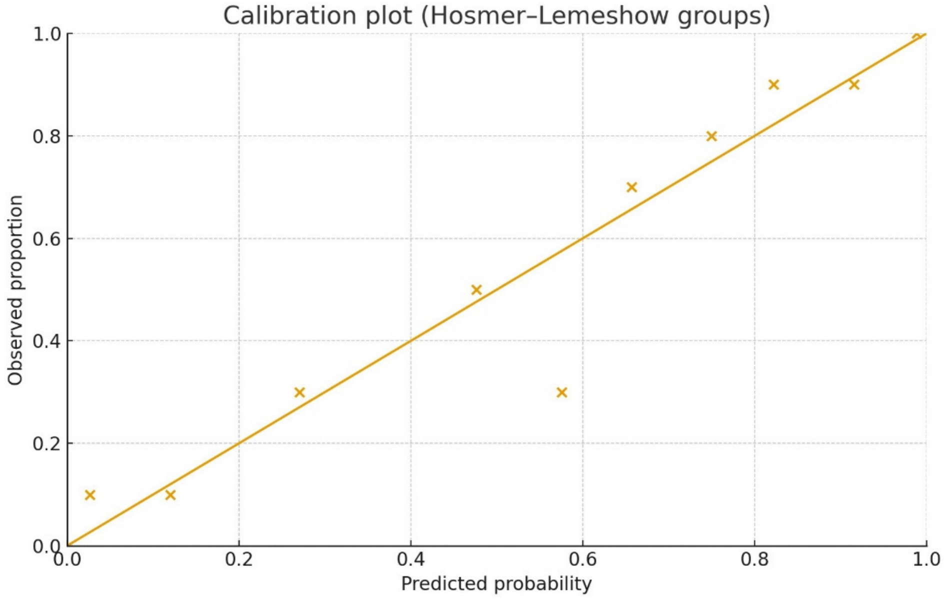

Calibration plot showing observed proportions of vascular reactivity dysfunction (y-axis) against model-predicted probabilities (x-axis) across 10 deciles of risk. The solid 45° line represents perfect calibration.

Supplementary Figure S2. Decision curve analysis for the leptin-based logistic model predicting vascular reactivity dysfunction.

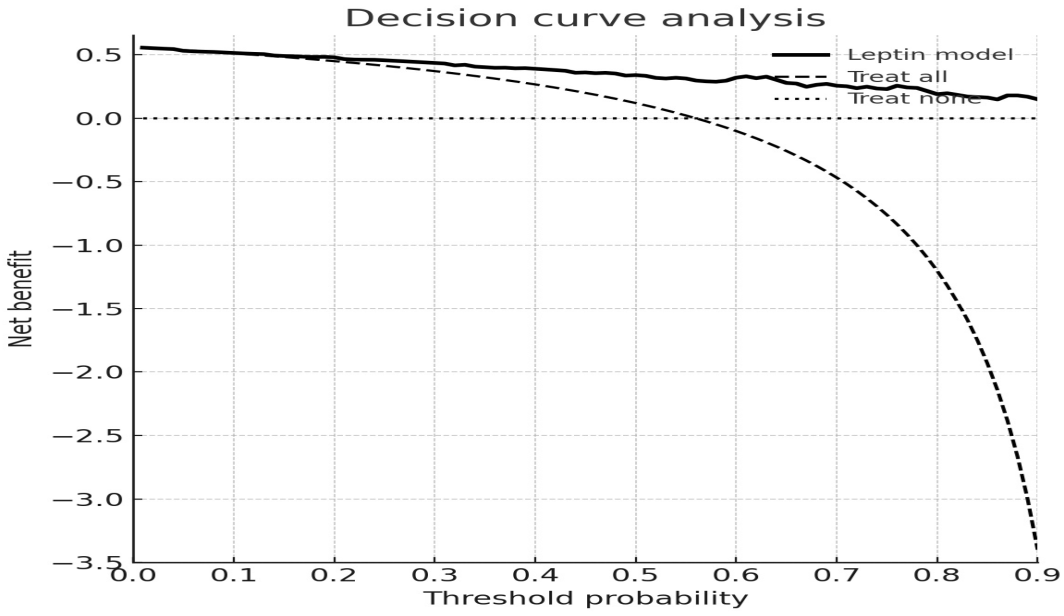

The leptin model (solid line) yields a greater net benefit than treating all patients (dashed line) or not treating any patients (dotted line) across threshold probabilities of approximately 0.05–0.65.
